# Evaluation of the efficacy and safety of inhaled magnesium sulphate in combination with standard treatment in patients with moderate or severe COVID-19: A structured summary of a study protocol for a randomised controlled trial

**DOI:** 10.1186/s13063-021-05032-y

**Published:** 2021-01-18

**Authors:** Guitti Pourdowlat, Seyed Ruhollah Mousavinasab, Behrooz Farzanegan, Alireza Kashefizadeh, Zohreh Akhoundi Meybodi, Maedeh Jafarzadeh, Shadi Baniasadi

**Affiliations:** 1 Chronic Respiratory Diseases Research Centre, National Research Institute of Tuberculosis and Lung Diseases (NRITLD), Shahid Beheshti University of Medical Sciences, Tehran, Iran; 2 Department of Clinical Pharmacy, Faculty of Pharmacy, Shiraz University of Medical Sciences, Shiraz, Iran; 3 Critical Care Quality Improvement Research Centre, SBMU, Tehran, Iran; 4 Shahid Dr. Labbafinejad Hospital, Shahid Beheshti University of Medical Sciences, Tehran, Iran; 5 Shahid Sadoughi University of Medical Sciences, Yazd, Iran; 6 Tracheal Diseases Research Centre, National Research Institute of Tuberculosis and Lung Diseases (NRITLD), Shahid Beheshti University of Medical Sciences, Tehran, Iran

**Keywords:** COVID-19, hypoxia, inhalation, magnesium sulphate, protocol, randomised controlled trial, respiratory tract symptoms

## Abstract

**Objectives:**

Basic and clinical studies have shown that magnesium sulphate ameliorates lung injury and controls asthma attacks by anti-inflammatory and bronchodilatory effects. Both intravenous and inhaled magnesium sulphate have a clinical impact on acute severe asthma by inhibition of airway smooth muscle contraction. Besides, magnesium sulphate can dilate constricted pulmonary arteries and reduce pulmonary artery resistance. However, it may affect systemic arteries when administered intravenously. A large number of patients with covid-19 admitted to the hospital suffer from pulmonary involvement. COVID-19 can cause hypoxia due to the involvement of the respiratory airways and parenchyma along with circulatory impairment, which induce ventilation-perfusion mismatch. This condition may result in hypoxemia and low arterial blood oxygen pressure and saturation presented with some degree of dyspnoea and shortness of breath. Inhaled magnesium sulphate as a smooth muscle relaxant (natural calcium antagonist) can cause both bronchodilator and consequently vasodilator effects (via a direct effect on alveolar arterioles in well-ventilated areas) in the respiratory tract. We aim to investigate if inhaled magnesium sulphate as adjuvant therapy to standard treatment can reduce ventilation-perfusion mismatch in the respiratory tract and subsequently improve arterial oxygen saturation in hospitalized patients with COVID-19.

**Trial design:**

A multi-centre, open-label, randomised controlled trial (RCT) with two parallel arms design (1:1 ratio)

**Participants:**

Patients aged 18-80 years hospitalized at Masih Daneshvari Hospital and Shahid Dr. Labbafinejad hospital in Tehran and Shahid Sadoughi Hospital in Yazd will be included if they meet the inclusion criteria of the study. Inclusion criteria are defined as 1. Confirmed diagnosis of SARS-CoV-2 infection based on polymerase chain reaction (PCR) of nasopharyngeal secretions or clinical manifestations along with chest computed tomography (chest CT) scan 2. Presenting with moderate or severe COVID-19 lung involvement confirmed with chest CT scan and arterial oxygen saturation below 93% 3. Length of hospital stay ≤48 hours.

Patients with underlying cardiovascular diseases including congestive heart failure, bradyarrhythmia, heart block, the myocardial injury will be excluded from the study.

**Intervention and comparator:**

Participants will be randomly divided into two arms. Patients in the intervention arm will be given both standard treatment for COVID-19 (according to the national guideline) and magnesium sulphate (5 cc of a 20% injectable vial or 2 cc of a 50% injectable vial will be diluted by 50 cc distilled water and nebulized via a mask) every eight hours for five days. Patients in the control (comparator) arm will only receive standard treatment for COVID-19.

**Main outcomes:**

Improvement of respiratory function and symptoms including arterial blood oxygen saturation, dyspnoea (according to NYHA functional classification), and cough within the first five days of randomization.

**Randomisation:**

Block randomisation will be used to allocate eligible patients to the study arms (in a 1:1 ratio). Computer software will be applied to randomly select the blocks.

**Blinding (masking):**

The study is an open-label RCT without blinding.

**Numbers to be randomised (sample size):**

The trial will be performed on 100 patients who will be randomly divided into two arms of control (50) and intervention (50).

**Trial Status:**

The protocol is Version 5.0, January 05, 2021. Recruitment of the participants started on July 30, 2020, and it is anticipated to be completed by February 28, 2021.

**Trial registration:**

The trial was registered in the Iranian Registry of Clinical Trials (IRCT) on July 28, 2020. It is available on https://en.irct.ir/trial/49879. The registration number is IRCT20191211045691N1.

**Full protocol:**

The full protocol is attached as an additional file, accessible from the Trials website (Additional file 1). In the interest of expediting the dissemination of this material, the familiar formatting has been eliminated; this Letter serves as a summary of the key elements of the full protocol.

**Supplementary Information:**

The online version contains supplementary material available at 10.1186/s13063-021-05032-y.

## Supplementary Information

**Additional file 1.**

## Data Availability

We declare that the chief investigator and the corresponding author will have access to the final trial dataset. They will consider reasonable requests for data sharing. The requests can be sent to pourdowlat_g@yahoo.com.

