# Supplementary material for: Evaluation of the efficacy and safety of inhaled magnesium sulphate in combination with standard treatment in patients with moderate or severe COVID-19: A structured summary of a study protocol for a randomised controlled trial
Source: Trials. 2021 Jan 18;22:60. doi: 10.1186/s13063-021-05032-y (PMC7812339; doi:10.1186/s13063-021-05032-y)
Supplement: Supplementary file 1 — Additional file 1. [file 13063_2021_5032_MOESM1_ESM.pdf]

Evaluation of the efficacy and safety of inhaled magnesium sulphate in combination with standard treatment in patients with COVID-19: A structured summary of a study protocol for a randomised controlled trial

Guitti Pourdowlat[1], Seyed Ruhollah Mousavinasab[2], Behrooz Farzanegan[1], Alireza Kashefizadeh[3], Zohreh Akhoundi Meybodi[4], Maedeh Jafarzadeh[1], Shadi Baniasadi[5]

[1] Chronic Respiratory Diseases Research Center, National Research Institute of Tuberculosis and Lung Diseases (NRITLD), Shahid Beheshti University of Medical Sciences, Tehran, Iran.

[2] Department of Clinical Pharmacy, Faculty of Pharmacy, Shiraz University of Medical Sciences, Shiraz, Iran.

[3] Shahid Dr. Labbafinejad hospital, Shahid Beheshti University of Medical Sciences, Tehran, Iran.

[4] Shahid Sadoughi University of medical sciences, Yazd, Iran.

[5] Tracheal Diseases Research Center, National Research Institute of Tuberculosis and Lung Diseases (NRITLD), Shahid Beheshti University of Medical Sciences, Tehran, Iran.

Corresponding Author

Shadi Baniasadi, PharmD, PhD, Tracheal Diseases Research Center, National Research Institute of Tuberculosis and Lung Diseases, Masih Daneshvari Hospital, Daarabad, Niavaran, Tehran, Iran. Tel/Fax: +98-21 26105387, Email: sbaniasadi@yahoo.com, baniasadi@sbmu.ac.ir

Abstract

Due to the COVID-19 disease pandemic and its high mortality rate, finding an effective treatment for this disease is critical. Magnesium sulphate has anti-inflammatory, vasodilatory, and bronchodilatory effects and may be effective in the treatment of this disease. This study is a prospective, randomized, controlled and open-label trial that will be performed on 100 inpatients aged 18 to 80 years with a diagnosis of COVID-19 based on polymerase chain reaction (PCR) of nasopharyngeal secretions or chest computed tomography (chest CT) scan along with clinical manifestations. Patients will be randomly assigned to two groups. The intervention group will receive both standard treatment for COVID-19 (according to the national guideline) and magnesium sulfate inhalation and the control group will only receive standard treatment for COVID-19.

The primary outcomes are defined as improving respiratory function and symptoms including oxygen saturation, dyspnoea (according to NYHA functional classification), and cough. Secondary outcomes are evaluated as improving laboratory test results including C-reactive protein, the total number of lymphocytes, d-dimer, and ferritin.

چکیده

بررسی اثربخشی و ایمنی داروی منیزیم سولفات استنشاقی در ترکیب با درمان استاندارد در بیماران مبتلا به کووید-۱۹: یک کارآزمایی کنترل شده تصادفی

با توجه به پاندمی بیماری کووید-۱۹، میزان زیاد مرگ و میر ناشی از آن و اهمیت رسیدن به درمان موثر برای درمان این بیماری و احتمال موثر بودن منیزیم سولفات باتوجه به مکانیسم های مختلف این دارو (ضد التهاب، گشاد کننده عروق و گشاد کننده ریه) که در درمان این بیماری می تواند موثر باشد مطالعه ی حاضر طراحی گردید. این مطالعه به صورت یک کارآزمایی آینده نگرکنترل شده تصادفی و بدون کور سازی بوده و روی ۱۰۰ بیمار بستری ۱۸ تا ۸۰ سال با تشخیص بالینی کووید- ۱۹بر اساس نتایج واکنش زنجیره‌ای پلیمراز از ترشحات نازوفارنکس یا تظاهرات بالینی و یافته های سی تی اسکن انجام خواهد گرفت. بیماران به صورت تصادفی در یکی از دو گروه مداخله شامل درمان استاندارد کشوری (درمان استاندارد در فاز ریوی متوسط و شدید) به همراه منیزیم سولفات یا گروه کنترل شامل درمان استاندارد کشوری وارد خواهند شد .

اهداف اولیه این مطالعه شامل تعیین میزان اثربخشی منیزیم سولفات استنشاقی در بهبود علایم تنفسی بالینی شامل تنگی نفس و سرفه، و افزایش میزان اشباع اکسیژن خون شریانی و اهداف ثانویه شامل تعیین میزان اثربخشی منیزیم سولفات استنشاقی در بهبود علایم آزمایشگاهی شامل دی دایمر، پروتئین واکنشی سی، فریتین و تعداد لنفوسیتها می باشد.

مقدمه

کرونا ویروس ها ویروس های آران‌ای دار هستند که بخاطر اسپایک های تاج مانندی روی سطح خود اینگونه نامگذاری شده اند. (۱, ۲) کرونا ویروس ها پاتوژن های مهمی در انسان و مهره داران هستند که می توانند منجر به عفونت سیستم تنفسی، گوارشی و سیستم اعصاب مرکزی در انسان، دام، پرندگان، خفاش، موش و تعداد زیادی از حیوانات دیگر شوند (۲). شیوع سندروم حاد و شدید تنفسی در سال ۲۰۰۳/۲۰۰۲ تحت عنوان سارس و سندروم تنفسی خاورمیانه در سال ۲۰۱۲ تحت عنوان مرس نشان دهنده ی این است که امکان انتقال حیوان به انسان و انسان به انسان کرونا ویروس های جدید و نوظهور وجود دارد (۲). شیوع پنومونی بدون علت مشخص در ووهان چین در دسامبر ۲۰۱۹ توجه زیادی را در سراسر جهان جلب

کرد (۲, ۳). عامل ایجادکننده ی این پنومونی یک کرونا ویروس جدید بود که در ۵ آزمایشگاه مستقل تشخیص داده شد. در ۱۲ ژانویه ۲۰۲۰ سازمان بهداشت جهانی آن را به عنوان کرونا ویروس جدید ۲۰۱۹ نام گذاری کرد (۲). در ۱۱ فوریه ۲۰۲۰، سازمان بهداشت جهانی آن را کووید-۱۹ نام گذاری کرد (۳). سارس از آنزیم تبدیل کننده آنژیوتانسین ۲ به عنوان رسپتور استفاده می کند و در درجه ی اول سلول های اپی تلیال مژک دار برونش و پنوموسیت های تیپ دو را آلوده و درگیر می کند. مرس از دی پپتیدیل پپتیداز ۴ به عنوان رسپتور استفاده کرده و سلول ها ی اپی تلیال فاقد مژک برونش و پنوموسیت های تیپ دو را درگیر می کند (۴). در بیماران مبتلا به کووید-۱۹ تعداد لکوسیت ها در سیستم تنفسی به شکل غیر طبیعی نرمالی بالاتر از حد است. بیماری زایی اصلی کووید-۱۹ هدف قرار دادن سیستم تنفسی و ایجاد پنومونی شدیدی است. به طور قابل ملاحظه ای سطح خونی بالای سایتوکاین ها و کموکاین ها در بیماران مبتلا به کووید-۱۹ دیده می شود. مطالعات گذشته نشان داده است که ویروپورین ای که جزئی از ویروس است کانال های یونی جهت نفوذ کلسیم را تشکیل می دهد و همچنین اینفلامازوم NLRP3 را فعال می کند. (۵).

یکی از مشکلاتی که به دنبال ابتلا به بیماری کووید -۱۹ به وجود می آید و می تواند منجر به مرگ و میر شود بحث تنگی نفس، اختلال در تنفس بیمار و کاهش سطح اشباع اکسیژن شریانی میباشد (۶) . منیزیم سولفات دارای ویژگی های مختلف از جمله ویژگی های ضد التهابی (مانندکاهش میزان تولید تومور نکروزیس فاکتور آلفا و اینترلوکین ۶ که دو فاکتور التهابی مهم در پیشرفت بیماری کووید-۱۹ می باشند) می باشد که در مطالعات مختلف بررسی و اثبات گردیده است. با توجه به آنچه در بالا نیز ذکر گردید و در ادامه به آن پرداخته می شود به علت کاهش میزان تولید تومور نکروزیس فاکتور آلفا و اینترلوکین ۶ (۳) و همچنین ریلکس کردن عضلات صاف برونش ها و کاهش مقاومت عروقی پولمونر و بهبود ونتیلاسیون و پرفیوژن می تواند در درمان بیماران مبتلا به کووید-۱۹ به ویژه در مورد طوفان سایتوکاینی و گرفتاری سیستم تنفسی ایجاد شده در این بیماری موثر باشد. براساس مکانیسم های عملکردی منیزیم سولفات و پاتوژنز کوید-۱۹ می توان به نتیجه رسید

که از این دارو می توان به عنوان یکی از گزینه های پیشنهادی درمان کوید-۱۹ به ویژه در درمان مشکلات تنفسی ناشی از این بیماری استفاده نمود.

بررسی متون

منیزیم یکی از فراوان ترین کاتیون ها در بدن بوده و دارای کاربردهای بالینی فراوان می باشد. بیشتر یون منیزیم به صورت یون داخل سلولی وجود داشته و از کل منیزیم در بدن ۵۳ ٪ در استخوان، ۲۷ ٪ در بافت عضلانی، ۱۹٪ در بافت نرم و ۰.۵ ٪ در گلبول های قرمز و ۰.۳٪ در سرم توزیع می شود نیمی از این مقدار منیزیم به صورت آزاد وجود دارد و به آلبومین متصل نمی شود.(۷). منیزیم سولفات در اشکال دارویی تزریقی (عضلانی و وریدی) و فرم خوراکی وجود دارد. جذب این دارو در صورت مصرف به صورت خوراکی آهسته و ناچیز می باشد. شروع اثر این دارو سریع، یک ساعت و نیم تا شش ساعت به ترتیب در صورت تجویز به صورت داخل عروقی، تزریق عضلانی و خوراکی بوده و عمدتاً به آلبومین به میزان ۳۰ درصد اتصال می یابد. هر یک گرم از منیزیم سولفات حاوی ۹۸.۷ میلی گرم منیزیم المنتال می باشد (۸،۹،۱۰،۱۱،۱۲). فرم استنشاقی از ویال تزریقی این دارو به دست می آید. یون منیزیم به صورت مستقیم و غیر مستقیم در پروسه های فیزولوژیکی بدن نقش داشته و یک کوفاکتور اساسی برای واکنش های آنزیمی در بدن می باشد. یون منیزیم بسیاری از آنزیم هایی که در متابولیسم انرژی در بدن نقش دارند را فعال کرده و همچنین آنتاگونیست کلسیم با مکانیسم تنظیم کلسیم داخل سلولی می باشد. در مطالعه ای که توسط پناهی و همکارانش در مورد نقش منیزیم سولفات در بیماران بستری در آی سی یو انجام شد، مشاهده گردید که منیزیم نقش بسیار مهمی در بیماران بستری در آی سی یو دارد. منیزیم برای اصلاح هایپومنیزیمی در بیماران بستری در آی سی یو نقش بسیار حیاتی داشته و باعث کاهش مرگ و میر و کاهش مدت زمان بستری در آی سی یو می شود. (۷). سوگیموتو و همکاران او در مطالعه ای که انجام دادند دریافتند که تولید تومور نکروزیس فاکتور آلفا و اینترلوکین ۶ در مادران باردار به دنبال تجویز منیزیم سولفات کاهش یافت. آن ها همچنین مشاهده نمودند که به دنبال منیزیم سولفات تولید سایتوکاین در مادران باردار در حین زایمان کاهش یافته که به علت افزایش غلظت یون منیزیم می باشد. منیزیم سولفات نقش مهمی در فعال شدن فاکتور هسته ای کاپا بی و تولید سایتوکاین ایفا می کند (۱۳). در متاآنالیزی که توسط شان و

همکاران و در مورد اثر بخشی استفاده منیزیم سولفات به صورت تزریقی و نبولایزر برای درمان حملات حاد آسم بر روی ۱۷۵۴ بیمار کودک و بزرگسال انجام گرفت مشاهده گردید که تجویز منیزیم سولفات به صورت تزریقی و نبولایزر در ترکیب با بتا -۲ آگونیست ها و استروئیدهای سیستمیک باعث بهبود عملکرد ریه و کاهش میزان بستری شده بیماران با مکانیسم ریلکس کردن عضلات صاف برونش ها می شود (۱۴). در یک مطالعه که توسط مجتهدزاده و همکاران وی در آی سی یو بیمارستان سینا دانشگاه علوم پزشکی تهران برای یررسی موثر بودن تجویز منیزیم سولفات بر روی سطح سرمی اینترلوکین ۶ و پروتئین واکنشی سی انجام گرفت تعداد ۱۸بیمار بعد از جراحی انوریسم آئورت شکمی بررسی شدند و مشاهده گردید که تجویز منیزیم سولفات می تواند باعث ساپرس شدن اینترلوکین ۶ شود (۱۵). ساران و همکاران وی در مطالعه ای که در مورد اثر بخشی نبولایزر منیزیم سولفات به تنهایی و در ترکیب با سالبوتامول انجام دادند نتیجه گرفتند که نبولایزر منیزیم سولفات به تنهایی یا در ترکیب با سابوتامول دارای اثرات شاخص بالینی گشاده کننده برونش در آسم حاد بوده و باعث بهبود بالینی و افزایش پیک میزان جریان بازدم، کاهش ریت تنفس و کاهش ضربان قلب می گردد (۱۶). نتایج به دست آمده از مطالعه ای که توسط سان و همکاران وی در محیط آزمایشگاه انجام گرفت نشان داد که منیزیم ماکروفاژ را از فنوتیپ M0 به M2 تغییر داده و باعث کاهش فعال شدن فاکتور هسته ای کاپا بی شده و می تواند به عنوان یک عامل ضد التهابی جدید برای ساپرس کردن عوامل التهابی در کاربردهای بالینی استفاده گردد (۱۷). سولفات منیزیم می تواند سبب اتساع عروق ریه و کاهش مقاوت عروق ریوی شود گرچه وقتی به شکل تزریقی مصرف شود بر عروق سیستمیک نیز اثر می گذارد. با توجه به اینکه سولفات منیزیم به فرم استنشاقی می تواند همزمان به عنوان برونکودیلاتور و وازودیلاتور در ریه عمل کند مصرف آن در بیماران کووید-۱۹ می تواند ونتیلاسیون و پرفیوژن را بهبود بخشد و در نتیجه باعث افزایش اشباع اکسیژن خون شریانی شود.

اهداف کلی طرح:

بررسی اثربخشی و ایمنی داروی منیزیم سولفات استنشاقی در ترکیب با درمان استاندارد در بیماران مبتلا به کووید-۱۹

اهداف اولیه:

- تعیین میزان اثربخشی منیزیم سولفات استنشاقی در بهبود علایم تنفسی بالینی شامل تنگی نفس و سرفه بیماران کووید-۱۹

تعیین میزان اثربخشی منیزیم سولفات استنشاقی در افزایش اشباع اکسیژن خون شریانی بیماران کووید-۱۹

اهداف ثانویه:

- تعیین میزان اثربخشی منیزیم سولفات منیزم استنشاقی در بهبود علایم آزمایشگاهی بیماران کووید-۱۹

- تعیین میزان اثربخشی منیزیم سولفات استنشاقی در کاهش مورتالیتی بیماران کووید-۱۹

- تعیین میزان اثربخشی منیزیم سولفات استنشاقی در بهبود عملکرد ریوی بیماران کووید-۱۹

اهداف کاربردی طرح:

براساس مکانیسم های عملکردی منیزیم سولفات و پاتوژنز کووید-۱۹ می توان به این نتیجه رسید که از این دارو می توان به عنوان یکی از گزینه های پیشنهادی درمان به ویژه در درمان مشکلات تنفسی ناشی از این بیماری در کنار درمان استاندارد و همچنین تسریع بهبود و کاهش هزینه های بیماران مبتلا به کووید-۱۹ استفاده نمود.

فرضیات یا سوالات پژوهش (باتوجه به اهداف طرح):

- داروی منیزیم سولفات در بهبود علایم تنفسی (تنگی نفس و سرفه) بیماران کووید-۱۹ موثر است

- داروی منیزیم سولفات در بهبود و افزایش اشباع اکسیژن خون شریانی بیماران کووید-۱۹ موثر است

- داروی منیزیم سولفات در بهبود عملکرد ریوی بیماران کووید-۱۹ موثر است

- داروی منیزیم سولفات در بهبود یافته های آزمایشگاهی مانند دی دایمر، پروتئین واکنشی سی، فریتین و تعداد لنفوسیتها در بیماران کووید-۱۹ موثر است

- داروی منیزیم سولفات در مدت زمان بستری بیماران کووید-۱۹ در بیمارستان موثر است

- داروی منیزیم سولفات در کاهش نیاز بیماران کووید-۱۹ به بستری شدن در آی سی یو موثر است

- داروی منیزیم سولفات در کاهش مورتالیتی بیماران کووید-۱۹ موثر است

طراحی مطالعه و نحوه مداخله

این مطالعه یک مطالعه آینده نگر از نوع کنترل شده و تصادفی بوده و روی ۱۰۰ بیمار بستری ۱۸ تا ۸۰ سال با تشخیص بالینی کووید-۱۹ بر اساس واکنش زنجیره‌ای پلیمراز از ترشحات نازوفارنکس یا تظاهرات سی تی اسکن انجام خواهد گرفت. بیماران به صورت تصادفی به روش بلوک های جایگشتی در یکی از دو گروه مداخله (درمان استاندارد کشوری به همراه منیزیم سولفات) یا کنترل (درمان استاندارد کشوری) وارد خواهند شد (درهر گروه ۵۰ نفر قرار خواهند گرفت). بیمارانی که دارای کرایتریاهای زیر باشند وارد مطالعه خواهند شد ۱- امضا کردن فرم رضایت آگاهانه ۲- حداقل سن ۱۸ و حداکثر سن ۸۰ سال ۳- کمتر یا مساوی ۴۸ ساعت بستری در بیمارستان. بیمارانی که کرایتریاهای زیر را داشته باشند از مطالعه خارج خواهند شد؛ ۱- بیماران دارای بیماری بلاک قلبی ۲- بیماران با آسیب به میوکارد ۳- بیماران با برادی کاردی. تمامی بیمارانی که وارد این مطالعه می شوند فرم رضایت نامه را پر کرده و امضا خواهند کرد. برای کسب اطلاعات لازم در مورد شرایط بیماران پرسشنامه ای طراحی شده و در اختیار بیماران قرار داده می شود که تمامی سوالات در زمینه شرایط ورود و خروج پرسیده می شود و بر اساس آن و مشاوره با پزشک برای ورود یا خروج بیماران تصمیم گیری می شود و سپس بیماران به صورت تصادفی در یکی از دوگروه مداخله یا کنترل به صورت تصادفی قرارخواهند گرفت. بیماران در گروه مداخله در روز های یکم تا پنجم، هر ۸ ساعت یکبار، منیزیم سولفات به صورت استنشاقی (۵ سی سی از ویال ۲۰ درصد تزریقی یا ۲ سی سی از ویال ۵۰ درصد تزریقی که با ۵۰ سی سی آب مقطر رقیق شده و نبولایز می شود) در کنار درمان استاندارد دریافت می نمایند. بیماران در گروه کنترل درمان استاندارد دریافت می نمایند.

بررسی پیامد ها

پیامدهای اولیه:

۱- بهبود علایم تنفسی شامل تنگی نفس و سرفه: که درروزهای اول تا پنجم و در پایان هفته ی اول بستری با استفاده از پرسشنامه طراحی شده ارزیابی می شود.

۲- اشباع اکسیژن خون شریانی: درست لحظه ی قبل و یک ساعت بعد از هربار تجویز منیزیم سولفات توسط دستگاه پالس اکسیمتری اندازه گیری می شود.

پیامدهای ثانویه:

۱- پروتئین واکنشی سی: در روزهای یکم تا پنجم روزانه توسط آزمایشگاه اندازه گیری می شود.

۲- تعداد کل لنفوسیتها: درروزهای یکم تا پنجم روزانه توسط آزمایشگاه اندازه گیری می شود.

۳- دی دایمر: درروزهای یکم تا پنجم روزانه توسط آزمایشگاه اندازه گیری می شود.

۴- فریتین: درروزهای یکم تا پنجم روزانه توسط آزمایشگاه اندازه گیری می شود.

۵- عوارض جانبی و عدم تحمل منیزیم استنشاقی: توسط فرم عوارض جانبی جمع آوری خواهد شد.

روش محاسبه حجم نمونه

در منابع قبلی مطالعه کاملی که با تکیه بران بتوان حجم نمونه محاسبه کردد وجود ندارد اما اگر بخواهیم نمره بهبود کلی را که از علایم بیمار بدست می اید به اندازه   ۴ نمره کاهش دهیم و با در نظرگرفتن حداکثر انحراف معیار این نمره به اندازه ۵ تعداد ۵۰ بیمار برای هر گروه موردنیاز است.

سطح معنی داری ۵ درصد و توان ازمون ۸۰ درصد در نظر گرفته شده است .

$$n = \frac{(Z_{(1-\frac{\alpha}{2})} + Z_{1-\beta})^2 2S^2}{(\mu_1 - \mu_2)^2}$$

بیماران به صورت تصادفی در دو گروه منیزیم سولفات و درمان استاندارد و یا درمان استاندارد تقسیم بندی می شوند.

در ابتدای ورود به مطالعه، بیماران به صورت تصادفی به روش بلوک های جایگشتی در یکی از دو گروه تقسیم شده وارد خواهند شد (هر گروه ۵۰ نفر).

روش تصادفی سازی بلوک

در تصادفی سازی بلوک جایگشتی چهارتایی تمامی بلوک های ممکن به صورت زیر چیده می شوند. بلوک ۱: ام سی ام سی، بوک ۲: ام ام سی سی، بلوک ۳: ام سی سی ام، بلوک ۴: سی سی ام ام، بلوک ۵: سی ام سی ام، بلوک ۶: سی ام ام سی. برای انتخاب ۱۰۰نفر به ۲۵ بلوک نیاز داریم. این بلوک ها را به تصادف از اعداد ۱ تا ۶ انتخاب می کنیم. با استفاده از نرم افزار یک عدد تصادفی بین اعداد ۱ تا ۶ انتخاب می کنیم. به عنوان مثال اگر شماره ۶ به عنوان بلاک اول و شماره ۲ به عنوان بلوک

دوم انتخاب شود، به افرادی که وارد مطالعه می شوند به ترتیب سی ام سی ام سی ام سی سی داده می شود. در نهایت گروه ام گروه منیزیم سولفات و گروه سی گروه کنترل می باشد.

ملاحظات اخلاقی

از تمامی بیماران قبل از ورود به مطالعه فرم رضایت آگاهانه اخذ خواهد شد. اطلاعات بیماران محرمانه باقی مانده و هیچ یک از مشخصات فردی افراد شرکت کننده از جمله اسم و فامیل آنها وارد کامپیوتر نخواهد شد و به تمامی افراد یک کد اختصاصی داده شده و ورود داده ها و آنالیز بر اساس آن انجام خواهد شد. اطلاعات اولیه در محل امن و نزد مجری اصلی تا اتمام طرح و انتشار نتایج آن باقی خواهد ماند. در صورت تمایل بیمار به خروج از مطالعه بیمار می تواند از مطالعه خارج شود.

محدودیتهای اجرایی طرح و روش رفع آن ها

از محدودیت های این طرح نحوه ی تهیه فرم استنشاقی منیزیم سولفات می باشد که با فرمول ارائه شده (۵ سی سی از ویال ۲۰ درصد تزریقی یا ۲ سی سی از ویال ۵۰ درصد تزریقی که رقیق شده و نبولایز می شود) می توان از فرم تزریقی این فراورده، فرم استنشاقی آن را به دست آورد.

References

1. Corman VM, Muth D, Niemeyer D, Drosten C. Hosts and sources of endemic human coronaviruses. Adv Virus Res. 2018;100:163-188.
2. Chen Y, Liu Q, Guo D. Emerging coronaviruses: genome structure, replication, and pathogenesis. J Med Virol. 2020;92(4):418-423.
3. Rothan HA, Byrareddy SN. The epidemiology and pathogenesis of coronavirus disease (COVID-19) outbreak. J Autoimmun. 2020;109:102433.
4. Cui J, Li F, Shi Z-L. Origin and evolution of pathogenic coronaviruses. Nature reviews Microbiology. 2019;17(3):181-92.
5. Colchicine Efficacy in COVID-19 Pneumonia. https://clinicaltrials.gov/ct2/show/NCT04322565.
6. Xu Z, Shi L, Wang Y, Zhang J, Huang L, Zhang C, et al. Pathological findings of COVID-19 associated with acute respiratory distress syndrome. Lancet Respir Med. 2020;8(4):420-422.
7. Panahi Y, Mojtahedzadeh M, Najafi A, Ghaini MR, Abdollahi M, Sharifzadeh M, et al. The role of magnesium sulfate in the intensive care unit. EXCLI Journal 2017;16:464-482.
8. Magnesium sulfate: Drug information. Lexicomp 2020. https://www.lib.utdo.ir/contents/magnesium-sulfate-drug-information?search=magnesium%20sulphate&source=panel_search_result&selectedTitle=1~148&usage_type=panel&kp_tab=drug_general&display_rank=1. Accessed on December 2020.
9. Lu J, Pfister M, Ferrari P, Chen G, Sheiner L. Pharmacokinetic-Pharmacodynamic Modelling of Magnesium Plasma Concentration and Blood Pressure in Preeclamptic Women. Clin Pharmacokinet 2002;41(13): 1105-1113.
10. Schumacher SA. The pharmacokinetics and pharmacodynamics of intravenous magnesium sulfate in horses. Dissertation. The Ohio State University. 2019.
11. Lu J, Pfister M, Ferrari P, Chen G, Sheiner L. Pharmacokinetic-Pharmacodynamic Modelling of Magnesium Plasma Concentration and Blood Pressure in Preeclamptic Women. Clin Pharmacokinetic 2002;41(13):1105-1113.
12. Okusanya BO, Oladapo OT, Long Q, Lumbiganon P, Carroli G, Qureshi Z, et al. Clinical pharmacokinetic properties of magnesium sulphate in women with pre-eclampsia and eclampsia. BJOG. 2016;123(3):356–366.
13. Sugimoto J, Romani AM, Valentin-Torres AM, Luciano AA, Ramirez Kitc CM, Funderburg N, Mesiano S, Bernstein HB. Magnesium Decreases Inflammatory Cytokine Production: A Novel Innate Immunomodulatory Mechanism. J Immunol. 2012;188(12).
14. Shan Z, Rong Y, Yang W, Wang D, Yao P, Xie J, Liu L. Intravenous and nebulized magnesium sulfate for treating acute asthma in adults and children: A systematic review and meta-analysis. Respir Med. 2013;107(3):321-30.
15. Mojtahedzadeh M, Chelkeba L, Ranjvar- Shahrivar M, Najafi A, Moini M, Najmeddin F, Sadeghi K, Barkhordari KH, Gheymati A, Ahmadi A. Randomized Trial of the Effect of Magnesium Sulfate Continuous Infusion on IL-6 and CRP Serum Levels Following Abdominal Aortic Aneurysm Surgery. IJPR 2016;15(4):951-956.
16. Sarhan HA, EL-Garhy OH, Ali MA, Youssef NA. The efficacy of nebulized magnesium sulfate alone and in combination with salbutamol in acute asthma. Drug Des Devel Ther. 2016;10: 1927–1933.
17. Sun L, Li X, Xu M, Yang F, Wang W, Niu X. In vitro immunomodulation of magnesium on monocytic cell toward anti-inflammatory macrophages. Regenerative Biomaterials 2020;7(4):391–401.
